# Determinants of Lack of Access to Treatment for Women Diagnosed with Breast Cancer in Brazil

**DOI:** 10.3390/ijerph19137635

**Published:** 2022-06-22

**Authors:** Maria Nizete Tavares Alves, Maria de Fátima Vasques Monteiro, Fernanda Tavares Alves, Francisco Winter dos Santos Figueiredo

**Affiliations:** 1 Observatory of Health-Related Socioeconomic Inequalities, Inequities and Vulnerabilities, Centro Universitário FMABC, Santo André 09060-870, Brazil; nizeteta@gmail.com; 2 Grupo de Estudos em Direitos da Criança e do Adolescente e Juventude, Universidade Regional do Cariri, Crato 63105-010, Brazil; fatimavasmonteiro@gmail.com; 3 Médica da Estratégia de Saúde da Família, Juazeiro do Norte 63010-000, Brazil; fernandatavaresalves@hotmail.com; 4 Epidemiology Research Group, Instituto Tocantinense Presidente Antônio Carlos, ITPAC-Porto Nacional, Porto Nacional 77500-000, Brazil

**Keywords:** social vulnerability, epidemiology, public health, unique system of health

## Abstract

Access to timely treatment is essential for the probability of the cure and reduction of severe breast cancer cases. In Brazil, legislation states that cancer treatment must start within 60 days of diagnosis. This study analyzed the factors associated with lack of access to breast cancer treatment in women with a confirmed diagnosis inserted in the health system. We collected secondary data from Brazilian women with a diagnosis and without treatment from January to December 2019 through the Cancer Hospital Registers developed by the National Cancer Institute. Our findings indicate that most women (60.11%) are diagnosed with stage II cancer but are without treatment. Most of them are aged 18–70 years, non-white race/color, have a low educational level and are from the Southeast Brazilian region. In addition, social inequalities are determinant in women’s lack of access to breast cancer treatment.

## 1. Introduction

Breast cancer (BC) has a high incidence among women globally, with approximately 2.3 million new cases estimated in 2020. It is the most frequent cause of cancer death in the female population, with an estimated 684,996 fatal victims in 2020 [1]. It is also the type of cancer that mainly affects women in Brazil. In 2020 the estimate of the Instituto Nacional de Câncer José Alencar Gomes da Silva (INCA) was 66,000 new cancer cases for each year of the triennium 2020–2022.

The challenge is to ensure timely access to diagnosis and treatment in the face of increasing estimates of the number of cases in order to set up the appropriate treatment (radiotherapy or chemotherapy or surgeons) and, through a rehabilitation process, to reinsert the patients in their social and working context [2,3]. In Brazil, the right to access health services at all levels of care is guaranteed by the Organic Health Law No. 8.080 of 1990, regardless of social or personal characteristics, and it also guarantees integrality in the different care levels [4].

The National Oncology Care Policy (PNAO—Política Nacional de Atenção Oncológica) launched in 2005 through the Ordinance GM/MS 2439 reiterates that cancer is a public health problem, and from there, the Oncology Care Network was created. In 2012, a milestone in the treatment of cancer took place with Law No. 12,732, which ensures patients with malignant neoplasms initiate medical treatment within 60 days after diagnosis [5].

Besides all the advances in the Brazilian legislation, the inequities of the access to health services indicates that the fragility of health attention compromises the guarantee of equanimous caring integrality [6]. The expansion of primary attention increased the access to women, but still presents fragilities around assistance. There is already consensus in the literature that a smaller time between diagnosis and treatment results in better prognoses and greater chances of survival for the patient. In the more advanced stages, fast intervention is fundamental to treatment efficiency [7].

In Brazil, despite advances in screening, the lack of timely treatment impacts the increase of BC mortality rates due to social and regional disparities [8]. An increase of the incidence and mortality of BC has been observed in the last three decades, as a result of changes in demographic settings, urbanization, population aging and lifestyle changes [9]; this is associated with the lack of timely treatment and can have great impacts on patients’ health.

According to Instituto Nacional do Câncer [1], the incidence and mortality of cancer increases worldwide also due to the change of the distribution and prevalence of risk factors for cancer, mainly those associated with socioeconomic development.

It is observed that BC is a potentially curable disease; if diagnosed and treated in its initial stages, the prognosis is good [10]. However, there has been little debate about the social and economic factors that could influence access to treatment. Thus, this study aims to analyze the factors associated with the lack of access to BC treatment in women with a confirmed BC diagnosis recorded in the health system.

## 2. Materials and Methods

### 2.1. Study Design

Based on secondary data from the Cancer Hospital Registers (CHR), this is a cross-sectional study of Brazilian women with a confirmed BC diagnosis from 1 January to 31 December 2019.

### 2.2. Participants

Brazil is a country of continental territorial extension, divided into five administrative regions: North, Northeast, South, Southeast, and Center-West. Their social and cultural development characteristics make Brazil an extremely peculiar country concerning each region’s socioeconomic and health conditions. Non-analytical data were excluded, which according to the Ministry of Health/INCA, 2010, include records of cases that are less complete than analytical cases. They appear in the CHR Annual Report only with general information to document the hospital comprehensive care profile and their impact on hospital costs, including male case records, other types of cancer, and forms with incomplete data. Thus, we included data from patients with a confirmed diagnosis of BC and without treatment and data from patients without diagnosis and without treatment from January to December 2019; the analysis units were from the hospital records of the patients.

### 2.3. Data Source

Data were extracted from the CHR, a web-based system developed by the National Cancer Institute (INCA in Portuguese), available on the website http://irch.inca.gov.br/RHC/Net/, accessed on 7 August 2021. They were stored and managed in Microsoft Excel 2015 spreadsheets.

The CHR is responsible for collecting, storing, processing, and analyzing information from patients treated in a hospital unit with a confirmed cancer diagnosis. The data are collected systematically and allow patient follow-up to monitor and evaluate the quality of the service provided, including the outcomes of cancer treatment.

The CHR includes sociodemographic, clinical, and treatment characteristics in its records. It is mandatory to fill in and maintain data in the CHR in the High Complexity Oncology Care Units and Centers to comply with the requirements of the Ministry of Health [11].

Included were the records of cases of women who already had a confirmed diagnosis, but accessed the service without initiating the treatment (the causes of not-initiating the treatment can be because of patient refusal or referral to another health service, for example). Once the woman already had a confirmed diagnosis and was forwarded to another service, they formed the case group. The data of women who had their first contact with hospital services that had CHR and came to the service without diagnosis and without treatment formed the control group for the present study.

### 2.4. Variables

Sociodemographic data were extracted, such as age group (18–39 years old, 40–49 years old, 50–69 years old and 70 years old or more) and color (white or non-white—which included blacks, browns, indigenous people, and Quilombolas, to identify a racial vulnerability). Educational level (None, Elementary School, High school and Higher Education), marital status (Single, Married or Widow/Separate) and smoking status (current smoker, former smoker, and non-smokers) were also analyzed, as well as alcoholism (present alcoholism, former alcoholic, and non-alcoholic), region of residence (considering the five administrative areas of Brazil: North, Northeast, South, Southeast, and Midwest), clinical history, such as a family history of cancer (presence or absence), the origin of the patient’s referral from the Unified Health System (SUS), either not directed by SUS or on their own, and tumor stage at diagnosis (I, II, III and IV, according to the BI-RADS classification).

### 2.5. Ethical Aspects

This study analyzed secondary data available in the public system, where the patient is not identified. According to Resolution No. 510, from 7 April 2016, an informed consent form nor the approval of the ethics committee were requested [12].

### 2.6. Statistical Analysis

We performed a descriptive analysis of the study population’s characteristics using absolute and relative frequencies. Then, we used the Chi-square test and assessed factors associated with lack of access, odds ratio (OR) values with a 95% confidence interval (CI95%), and estimated respective p values through multivariate logistic regression. The significance level was 5%. The statistical software used was Stata (StataCorp, LC, College Station, TX, USA) version 11.0.

## 3. Results

We extracted 2525 BC cases reported in women from 1 January to 31 December 2019. The women were aged between 18 and 70 years at diagnosis. Most were non-white (53.27%), had a low education level (56.59%), lived in the Southeast Brazilian region (64.04%), and had BC stage II (35.41%).

**Figure 1:**
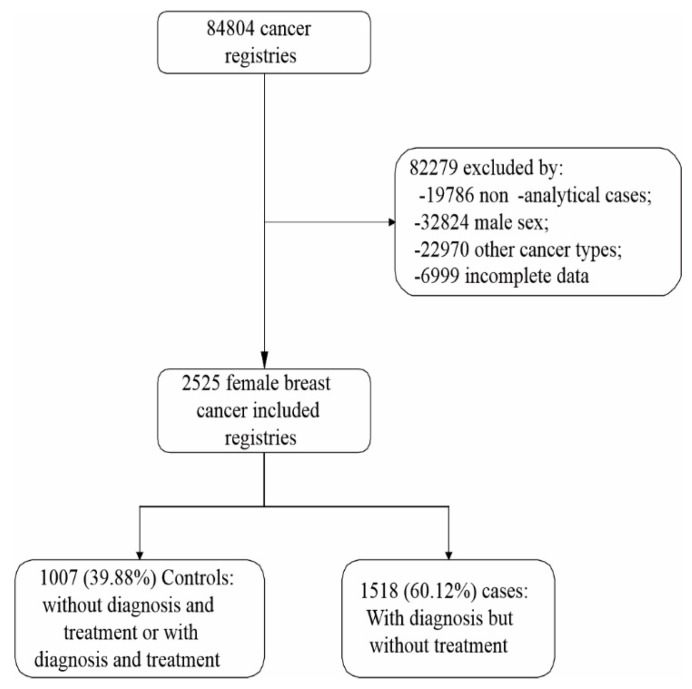
Flowchart for selecting the data included in this study.

Approximately 60% of BC hospital records were of women who already had a diagnosis but had not started treatment, representing the case group of the present study (Figure 1).

When analyzing the factors associated with the cases of women who arrived at the service with diagnosis and without treatment, age in the younger groups (18–39 years and 40–49 years, *p* = 0.001), not being white (*p* < 0.001), having higher levels of education (high school and higher education) (*p* = 0.003), residing in the North and Northeast regions (*p* < 0.001) and late diagnosis (stages III and IV) (*p* = 0.001) (Table 1) were observed as determinants.

When analyzing the factors associated with the barrier to starting treatment in women diagnosed with BC, it was observed that the age variable constitutes a barrier to accessing treatment; women aged 70+ years 53.66% (OR 0.56; CI 95% 0.76–0.97; *p* = 0.016) are less likely to have access than younger women. Race/skin color is also presented as a barrier to admission; non-white women 65.58% (OR 1.12; CI 95% 1.04–1.20; *p* = 0.002) have lower chances of accessing therapy than white-skinned women. Women with a better education at higher education level 69.23% (OR 1.33; CI 95% 1.13–1.57; *p* = 0.001), have lower chances of accessing treatment than women with low education. On the other hand, 42.08% of women residing in the South region (OR 073; CI 95% 0.59–0.89; *p* = 0.002) are less likely to access treatment. The analysis of BC stage at diagnosis showed that women with stage III (OR 1.12; CI 95% 1.02–1.23; *p* = 0.013) and stage IV (OR 1.19; CI 95% 1.07–1, 33; *p* = 0.001) are less likely to have access to treatment at an early stage of the disease (Table 2).

The multivariate analysis of factors associated with barriers to breast cancer treatment showed that the variables race/color (OR 1.7; CI 95% 1.44–2.01; *p* < 0.001), education (OR 2.36; CI 95% 1.56–3.60; *p* < 0.001), diagnostic stage III (OR 1.44; CI 95% 1.15–1.80; *p* = 0.001) and diagnostic stage IV (OR 1.58; CI 95% 1,18–2.11; *p* = 0.002), remained independently associated with the outcome. Other features analyzed did not present statistically significant differences (Table 3).

## 4. Discussion

Our results show that 2525 cases of confirmed and untreated BC were registered in 2019, being in the most part women aged between 18 and 70 years-old, non-white race/color, low schooling, residents of the Southeast region and diagnostic stage II-BC. The findings show that 60.11% of women diagnosed with BC had difficulties accessing treatment. The barriers observed were being aged between 18 and 49 years, of non-white race/color, a higher level of schooling, living in the North and Northeast regions, and diagnosed with BC advanced stages III and IV.

Confirmation of a cancer diagnosis causes an intense emotional reaction, as many people associate the diagnosis with death. A review study on delayed diagnosis and treatment of cancer points to lack of knowledge about the disease, low income, low education, fear and difficulty in accessing care as factors to justify the delay in diagnosis and treatment [13].

We identified five distinct factors associated with difficulties in accessing treatment for women diagnosed with BC. The obstacles to receiving care are more remarkable for the elderly (70+ years), which makes it more worrying since the aging of the population is already a reality in our country. Additionally, most women with better education, who are non-white, and at advanced stages of the disease (III and IV) also face more difficulties accessing treatment.

In line with our findings, the literature points to regional disparities as barriers to accessing BC therapy. Social inequality associated with individual characteristics such as education, race/color, income, among others, puts these populations at disadvantages compared to the south and southeast regions; as they have worse health conditions and difficulties accessing care services [6,14].

We have a country with continental dimensions, but we have a public, universal health system with capillarity in all municipalities. The difficulty in accessing cancer treatment on time becomes evident due to the lack of integration of services with Health Care Networks [15].

In addition, the number of patients in the highest stages evidences the fragility in screening actions, showing that the lack of access directly impacts all BC stages. A temporal series retrospective study (1998 to 2002 and 2008 to 2012) on BC mortality reported the same factors as those found in our study, corroborating the data presented here [16].

In assessing cancer risk, the World Report (2021) indicates that the socioeconomic gradient for cancer incidence may vary in magnitude and direction at different cancer sites. Still, cancer mortality is higher, and survival is lower in groups with low socioeconomic status and other disadvantaged groups such as ethnic and racial minorities [17].

A reality also experienced in developed countries, such as the United States, where although white women have a higher incidence of BC, African-American women are more likely to die from the disease and this disparity is a result of the opportunity to access health services and treatment [18].

As for the Brazilian territory, the lack of access to BC treatment is more prominent in the North, Northeast, and Central-West regions, areas with more vulnerable populations, higher poverty rates, more significant barriers to accessing health services, and where people are highly dependent on the public healthcare system [14].

It is worth noting that most women have a diagnosis but no treatment, showing that access to therapy remains a substantial barrier to overcome. An improvement in the screening index does not indicate a reduction in social inequalities. Reinforcing our findings, a cohort study of 204,130 cases in Brazil reported that the increase in BC screening had not positively impacted the reduction of BC mortality [19]. In addition to the target population’s low mammography coverage, there is an inadequacy in the segment of suspicious lesions and the absence of admission to surgical treatment [20].

It is essential to associate the treatment access obstacles with social determinants. Studies that analyze access to cancer screening show enhanced results in performing mammograms, but these improvements occur in patients with better incomes using private healthcare services [21,22,23].

In 2014, the Brazilian Ministry of Health established a period of up to 60 days to start treatment for cancer cases (Ordinance GM nº 1.220/2014). Even though it is established as a priority by ministerial decree, the treatment of BC is still not carried out promptly, even knowing that the relationship between diagnosis and time to initiate treatment has a direct impact on the quality of life and the survival of patients [19].

Souza et al., 2019 [6], in a study carried out in Piaui, Brazil, between 2016 and 2017, found that access to treatment remained delayed for more than 60 days in 71.6% of women diagnosed with BC. The same evidence was found in North Carolina, USA, between January 2000 and December 2002 in research that evaluated the impact of delayed treatment on survival after a confirmed BC diagnosis. One in ten women waited more than 60 days to start treatment, and that wait was associated with a 66% and 85% increased risk of overall and breast cancer-related death [24].

Another study from 2013 in the United States of America documented that those curative surgeries with intervals higher than 12 weeks are associated with increased BC mortality [25]. The study shows that having a confirmed diagnosis but not having started the treatment also represents a barrier to accessing treatment on time when inserted in the CHR.

The analysis carried out in 2019 from the CHR reports corroborates these data where the authors showed that patients “without diagnosis and treatment” were able to access treatment in less time than patients “with diagnosis and without treatment” [26].

Furthermore, not being white, having higher education and more advanced stages of diagnosis were the main determinants of access to treatment found in this study. Aligned with our results, a cross-sectional study of women with a confirmed diagnosis of primary BC in Belo Horizonte in 10 oncology units from 2010 to 2013 points out worse results for women of black race/color among women served in the public and private systems, showing that race and socioeconomic conditions contribute to women having worse health outcomes [27].

The level of schooling is a social determinant that impacts the understanding of the health-disease process. Still, we found that having more schooling did not positively impact access to treatment. The study by Buranello et al. [21] observed that women who undergo more screening tests have higher education, income > 2 MW, white race, are not family heads and are living with a partner. Oliveira et al. [28] indicated that women with a higher level of education were more likely to undergo mammography than those with a lower educational level. It reinforces that those social determinants are intrinsically related to barriers to accessing BC screening and treatment in Brazil.

Advanced stages are worrying because they indicate a fragility in access to treatment on time, which undoubtedly impacts these women’s quality of life, care and treatment costs. The study by Oliveira et al. [28], in line with our results, showed that despite creating a network to serve BC, we still find assistance voids that create barriers to access and compromise the quality of care. The same study points to a worrying situation: “approximately half of the municipalities did not refer patients for breast surgery in the public network and the SUS, 17.8% did not refer patients for chemotherapy and 49% for radiotherapy”.

Difficulties in accessing BC treatment for women using the SUS are still an essential barrier to reducing breast cancer morbidity and mortality. This study may have limitations inherent to using secondary data due to the weaknesses presented in health information systems and difficulties in accessing the internet in some regions of Brazil, which may influence the registration of patients in the CHR system. Nevertheless, we used the INCA database; the information was retrieved from the CHR, a vital data source, with updated records of BC treatment in Brazil.

It is important to emphasize this study’s positive points, such as the number of cases analyzed and the study results, to confront the difficulty of accessing BC treatment. Our findings support the need to develop strategies to face the lack of access from the reduction of inequalities in most vulnerable populations, the need for improvements in the oncology care network, and a better understanding of the lack of access for health professionals and managers to the public health system in Brazil.

## 5. Conclusions

Social inequalities directly affect access to treatment for women with a confirmed breast cancer diagnosis in Brazil. There is a need to think of differentiated strategies and to strengthen public policies to reduce social inequalities, strengthen the oncology network and reduce morbidity and mortality caused by breast cancer.

## Figures and Tables

**Table 1:** Univariate analysis of the factors associated with lack of access to treatment in diagnosed women.

Variable	Total *n* (%)	Control 1007 (39.9%)	Cases 1.518 (60.1%)	*p*-Value ^1^
Age Range (Years)				
18–39	284 (11.25)	89 (31.34)	195 (68.66)	0.001
40–49	558 (22.10)	217 (38.89)	341 (61.11)	
50–69	1273 (50.42)	511 (40.14)	762 (59.86)	
70+	410 (16.24)	190 (46.34)	220 (53.66)	
Ethnicity				<0.001
White	1180 (46.73)	544 (46.10)	636 (53.90)	
No White	1345 (53.27)	463 (34.42)	882 (65.58)	
Educational level				0.003
None	148 (5.86)	65 (43.92)	83 (56.08)	
Elementary School	1429 (56.59)	598 (41.85)	831 (58.15)	
High school	649 (25.70)	252 (38.83)	397 (61.17)	
Higher Education	299 (11.84)	92 (30.77)	207 (69.23)	
Marital status				0.660
Single	559 (22.14)	214 (38.28)	345 (61.72)	
Married	1402 (55.52)	568 (40.51)	834 (59.49)	
Widow/Separate	564 (22.34)	225 (39.89)	339 (60.11)	
Smoking				0.564
Never	1646 (71.60)	647 (39.31)	999 (60.69)	
Former smoker	379 (16.49)	142 (37.47)	237 (62.53)	
Current smoker	274 (11.92)	114 (41.61)	160 (58.39)	
Alcohol consumption				0.065
Never	1726 (77.16)	671 (38.88)	1055 (61.12)	
Former	134 (5.99)	42 (31.34)	92 (68.66)	
Current	377 (16.85)	161 (42.71)	216 (57.29)	
Region				<0.001
Central-West	83 (3.32)	29 (34.94)	54 (65.06)	
Northeast	311 (12.43)	104 (33.44)	207 (66.56)	
South	385 (15.38)	223 (57.92)	162 (42.08)	
Southeast	1603 (64.04)	612 (38.18)	991 (61.82)	
North	121 (4.83)	28 (23.14)	93 (76.86)	
Cancer family history				0.270
No	871 (37.80)	352 (40.41)	519 (59.59)	
yes	1433 (62.20)	546 (38.10)	887 (61.90)	
Health service reference				0.234
SUS	2175 (86.14)	855 (39.31)	1320 (60.69)	
No SUS	340 (13.47)	149 (43.82)	191 (56.18)	
Private	10 (0.40)	3 (30.00)	7 (70.00)	
Diagnostic stage				0.001
I	627 (24.83)	285(45.45)	342(54.55)	
II	894 (35.41)	367(41.05)	527(58.95)	
III	708 (28.04)	252(35.59)	456(64.41)	
IV	296 (11.72)	103(34.80)	193(65.20)	

^1^ Chi-square test.; SUS: *Sistema Único de Saúde.*

**Table 2:** Analysis of factors associated with barriers to initiation of treatment (treatment barrier) in women diagnosed with breast cancer.

Variable	Treatment Barrier OR (CI 95%)	*p*-Value ^1^
Age Range (Years)		
18–39	Ref	Ref
40–49	0.90 (0.82; 1.003)	0.059
50–69	0.93 (0.85; 1.02)	0.124
70+	0.56 (0.76; 0.97)	0.016
Ethnicity		
White	Ref	Ref
No White	1.12 (1.04; 1.20)	0.002
Educational level		
None	Ref	Ref
Elementary School	1.09 (0.94; 1.26)	0.254
High School	1.13 (0.97; 1.32)	0.123
Higher Education	1.33 (1.13; 1.57)	0.001
Region		
Central-West	Ref	Ref
Northeast	1.07 (0.89; 1.27)	0.467
South	0.73 (0.59; 0.89)	0.002
Southeast	1.02 (0.86; 1.20)	0.848
North	1.19 (0.99; 1.43)	0.067
Diagnostic stage		
I	Ref	Ref
II	1.07 (0.98; 1.17)	0.119
III	1.12 (1.02; 1.23)	0.013
IV	1.19 (1.07; 1.33)	0.001

^1^ Logistic Regression; OR: odds ratio; CI 95% confidence interval of 95%; Ref: reference category.

**Table 3:** Multivariate analysis of factors associated with lack of access to treatment in diagnosed women.

Variable	OR (CI 95%)	*p*-Value ^1^
Ethnicity		
White	Ref	Ref
No White	1.71 (1.44; 2.01)	<0.001
Educational level		
None	Ref	Ref
Elementary School	1.23 (0.87; 1.74)	0.238
High School	1.46 (1.01; 2.12)	0.041
Higher education	2.36 (1.56; 3.60)	<0.001
Diagnostic stage		
I	Ref	Ref
II	1.20 (0.98; 1.49)	0.077
III	1.44 (1.15; 1.80)	0.001
IV	1.58 (1.18; 2.11)	0.002

^1^ Logistic Regression; OR: odds ratio; CI 95% confidence interval of 95%; Ref: reference category.

## Data Availability

The data used in this study can be accessed on website http://irch.inca.gov.br/RHC/Net/ (accessed on 7 August 2021).
